# Abnormal Proteomics Profile of Plasma Reveals the Immunological Pathogenesis of Severe Aplastic Anemia

**DOI:** 10.1155/2022/3700691

**Published:** 2022-05-06

**Authors:** Weiwei Qi, Yu Zhang, Wenhao Yang, Chunyan Liu, Huaquan Wang, Rong Fu, Zonghong Shao

**Affiliations:** Department of Hematology, Tianjin Medical University General Hospital, 154 Anshan Street, Heping District, Tianjin 300052, China

## Abstract

Severe aplastic anemia (SAA) is an immune-mediated bone marrow failure characterized by pancytopenia. This study was aimed at uncovering proteins of plasma that were differentially expressed in SAA patients. 8 SAA patients and 8 health controls were enrolled and detected by data independent acquisition (DIA) technology. 154 differential expression proteins (DEPs) in plasma of SAA patients were identified. GO and KEGG analyses indicated DEPs were mainly involved in the immune system process. Specifically, C-C motif chemokine 18 (CCL18), matrix metalloproteinase-3 (MMP3), histidine-rich glycoprotein (HRG), and lactotransferrin (lactoferrin (Lf)) may play an important role in the immune pathogenesis of SAA. CCL18, MMP3, HRG, and Lf might be potential biomarkers for SAA.

## 1. Introduction

Severe aplastic anemia (SAA) is characterized by pancytopenia and bone marrow failure, which is considered immune-mediated destruction of hematopoietic cells caused by autoreactive T cells [1]. Currently, hematopoietic stem cell transplantation is the first therapeutic choice for young patients with a matched sibling donor; antithymocyte globulin (ATG) and cyclosporine (CsA) with or without thrombopoietin receptor agonist eltrombopag are considered a highly effective immunosuppressive strategy in old patients and a therapeutic option for younger patients without a matched sibling donor [2]. Because of the improvement of treatment, the 10-year overall survival of AA was greater than 80%. However, some patients died of serious infection or ineffective immunosuppressive therapy. Early diagnosis and evaluation of the patient's immune status are crucial to a favorable outcome. It is important to find a biomarker to assess the immune status of the disease and assist the diagnosis and treatment of SAA. Proteomics research, which is widely used in the life sciences, has promoted the analysis of molecular mechanisms and been beneficial to the diagnosis of disease at the protein level. This project applied the next generation of nonlabeled quantitative proteomics technology to complete the analysis. In the data independent acquisition (DIA) mode, it provides unparalleled proteomic coverage as well as highly repeatable accurate quantification of large amounts of protein in each sample. Our study used DIA technology to detect differentially expressed proteins (DEPs) of plasma between SAA patients and normal controls, trying to identify the biomarkers for SAA and provide assistance in diagnosis and treatment.

## 2. Materials and Methods

### 2.1. Patients

Eight untreated SAA patients were identified, who were hospitalized in the Hematology Department of General Hospital Tianjin Medical University from January 2018 to September 2018. SAA patients were diagnosed according to international AA Study Group Criteria. The characteristics of patients were shown in Table 1. They were not exposed to ionizing radiation, radioactive substances, benzene, organochlorine pesticides, drugs, etc. Patients were excluded if they had abnormal chromosomes or paroxysmal nocturnal hemoglobinuria (PNH) clones. In addition, we excluded patients with other autoimmune diseases and tumors. All of SAA patients accepted the standard immunosuppressive treatment of ATG and CsA, with or without eltrombopag (EPAG). There were 8 healthy volunteers in the normal control group. Their routine examinations of blood were all completely normal. The study was approved by the Ethics Committee of Tianjin Medical University. Informed written consent was obtained from all patients or their parents in accordance with the Declaration of Helsinki.

### 2.2. Sample Preparation

**Figure 1:**
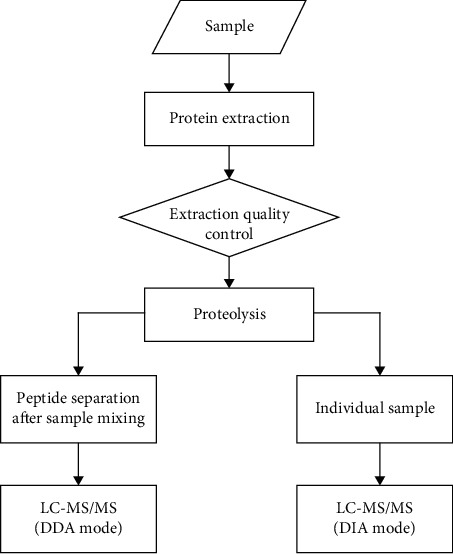
Graphic workflow of the present study.

All plasma samples were extracted from five milliliters fresh human peripheral blood which were collected in EDTA tubes, and all cases were collected at the time of diagnosis. After centrifugation at 3000 RPM for 10 min, plasma samples were collected and stored at −80°C until use. The flow chart of subsequent experiments was shown in Figure 1.

### 2.3. Protein Extraction, Quantitation, and Enzymatic Hydrolysis

Total protein was extracted in accordance with a standard protocol. In brief, samples were homogenized in lysis buffer containing PMSF, EDTA, and DTT. We oscillate with a tissue grinder for 2 minutes (power = 50 Hz, time = 120 s). Total protein was collected through centrifugation at 25,000 g^∗^4°C for 20 min. The supernatant was taken, and their concentration was determined using the Bradford method.

Then, trypsin enzyme was added in a ratio of protein: trypsin enzyme = 40 : 1, and the peptide was digested at 37°C. Enzymatic peptides were demineralized using a Strata X column and vacuumed to dryness.

### 2.4. High pH RP Separation and High-Performance Liquid Chromatography

All samples were taken 10 *μ*g, respectively, to mix, and the mixed 200 *μ*g was diluted with 2 mL of mobile phase A (5% ACN pH 9.8) and injected. The Shimadzu LC-20AB liquid phase system was used. The separation column was 5 *μ*m × 4.6 × 250 mm. The sample was subjected to liquid phase separation on a Gemini C18 column. Elution at a flow rate gradient of 1 mL/min: 5% mobile phase B (95% CAN, pH 9.8) for 10 minutes, 5% to 35% mobile phase B for 40 minutes, 35% to 95% mobile phase B for 1 minute, flow phase B lasted 3 minutes, and 5% mobile phase B equilibrated for 10 minutes. The elution peak was monitored at a wavelength of 214 nm, and one component was collected per minute, and the samples were combined with a chromatographic elution peak map to obtain 10 components, which were then freeze-dried.

The dried peptide samples were reconstituted with mobile phase A (2% ACN, 0.1% FA), centrifuged at 20,000 g for 10 minutes, and the supernatant was taken for injection. Separation was performed by Thermo UltiMate 3000 UHPLC. The sample was first enriched in a trap column and desalted and then serially connected to a self-loaded C18 column (150 *μ*m inner diameter, 1.8 *μ*m column size, and 25 cm column length) at a flow rate of 500 nL/min by the following effective gradient: 0-5 min, 5% mobile phase B (98% ACN, 0.1% FA); 5-160 min, mobile phase B linearly increased from 5% to 35%; 160-170 min, mobile phase B increased from 35% to 80%; 175 min, 80% mobile phase B; and 176-180 min, 5% mobile phase B. The nanoliter liquid phase separation end was directly connected to the mass spectrometer.

### 2.5. DDA Mass Spectrometry and Data Analysis

The peptides separated by liquid phase were ionized by a nanoESI source and then passed to a tandem mass spectrometer Q-Exactive HF (Thermo Fisher Scientific, San Jose, CA) for data-dependent acquisition (DDA) mode detection. Main parameter settings are as follows: ion source voltage was set to 1.6 kV, MS1 mass spectrometer scanning range was 350~1500 *m*/*z*, resolution was set to 60,000, MS2 starting *m*/*z* was fixed at 100, and resolution was 15,000. The secondary ion screening conditions for the secondary fragmentation were: charge 2+ to 7+, and the intensity of the peak intensity exceeding 10,000 was ranked in the top 20 parent ion. The ion fragmentation mode was HCD, and the fragment ions were detected in Orbitrap. The dynamic exclusion time was set to 30 s. The AGC was set to Level 3E6, Level1 1E5.

MaxQuant is a free protein identification and quantification software developed by Max Planck Institutes for high-precision mass spectrometry data. This project used the software to complete the identification of DDA data as a spectrum library for subsequent DIA analysis. In the operation, the original offline data was used as the input file, and the corresponding parameters and database were configured, and then, the identification and quantitative analysis were performed. The identification information that satisfies FDR ≤ 1% would be used to establish the final spectral library.

### 2.6. DIA Mass Spectrometry and Data Analysis

The peptide separated by liquid phase was ionized by a nanoESI source and then passed to a tandem mass spectrometer Q-Exactive HF (Thermo Fisher Scientific, San Jose, CA) for DIA mode detection. The main parameters were set: the ion source voltage was set to 1.6 kV, the MS 1 scan range was 350~1500 m/z, the resolution was set to 120,000, and the 350-1500 Da was divided into 40 windows for fragmentation and signal acquisition. The ion fragmentation mode was HCD, and the fragment ions were detected in Orbitrap. The dynamic exclusion time was set to 30 s. The AGC was set to Level1 3E6, Level2 1E5.

The DIA data was analyzed using Spectronaut, which used the iRT peptide to complete the correction for the retention time. In addition, Spectronaut integrated the mProphet scoring algorithm, which accurately reflected the matching of ion pairs. Then, based on the target-decoy model applicable to SWATH-MS, false positive control was performed with FDR 1%, thereby obtaining a significant quantitative result.

### 2.7. MSstats Difference Analysis and Bioinformatics Pipeline

MSstats is the R package from the Bioconductor repository. It can be used for statistical evaluation of significant differences in proteins or peptides of different samples and is widely used in targeted proteomics MRM, nonstandard quantitation, and SWATH quantitative experiments. The core algorithm is a linear mixed effect model (linear mixed effect models). The process preprocesses the data according to the set comparison group and then performs the significance test based on the model. In analysis for our experiment, differential protein screening was performed based on the fold change ≥ 2 and *P* value < 0.05 as the criterion for the significant difference. Finally, the functional analysis including GO and KEGG analyses of the DEPs were performed.

## 3. Results and Discussion

### 3.1. Identification of DEPs

**Figure 2:**
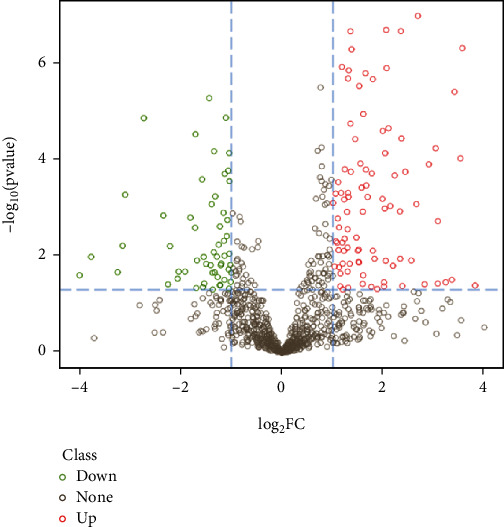
The volcano plot of the significantly dysregulated proteins between severe aplastic anemia (SAA) and normal. The *x*-axis of the graph is the protein difference multiple (take log_2_), and the *y*-axis is the corresponding −log_10_ (*P* value). In the figure, red dots represent 96 significantly upregulated protein, green dots mean 58 significantly downregulated protein, and gray dots are nonsignificantly altered protein.

DDA analysis was conducted to generate a spectral library; then, MaxQuant software was used to complete the search and identification. The MS/MS analysis identified a total of 6,558 distinct peptides resulting in the identification and quantification of 1,506 nonredundant proteins. These 1,506 proteins met the criterion that at least one unique peptide could be identified. We acquired mass spectral data for 16 samples by Q-Exactive HF (Thermo Fisher Scientific, San Jose, CA) instrument in DIA mode. Peptide and protein quantification were performed using Spectronaut and MSstats software. Using fold change ≥ 2 and *P* value < 0.05 as cutoffs, we found that a total of 154 proteins were significantly differentially expressed between SAA patients and normal controls. 96 of these proteins were upregulated, and the other 58 proteins were downregulated (Figure 2).

### 3.2. GO Analysis

**Figure 3:**
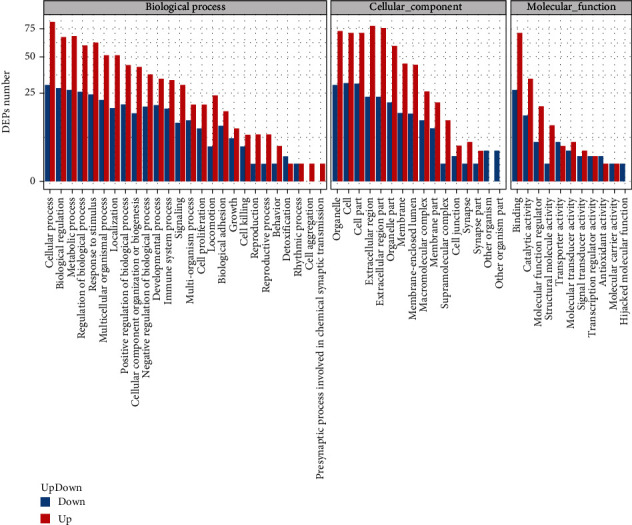
GO analysis of the differential expression proteins (DEPs) of severe aplastic anemia (SAA) vs. normal. The graph showed the biological processes, cellular component, and molecular function of DEPs based on GO classification, separately.

GO enrichment analysis was performed for these dysregulated proteins between SAA and normal groups in terms of 3 ontologies including biological process, cellular component, and molecular function (Figure 3). The SAA possesses significantly altered biological processes, such as cellular process, biological regulation, metabolic process, regulation of biological process, and response to stimulus and immune system process. Notably, there were 50 DEPs enriched in the immune system entries, such as C-C motif chemokine 18 (CCL18), lactotransferrin (lactoferrin (Lf)), and histidine-rich glycoprotein (HRG). In terms of cellular components, DEPs are mainly located in the organelle, cell, cell part, extracellular region, extracellular region part, organelle part, and membrane. GO analysis for molecular function revealed that these dysregulated proteins might play a role in binding, catalytic activity, molecular function regulator, structural molecule activity, transporter activity, molecular transducer activity, signal transducer activity, and transcription regulator activity.

### 3.3. KEGG Analysis

**Figure 4:**
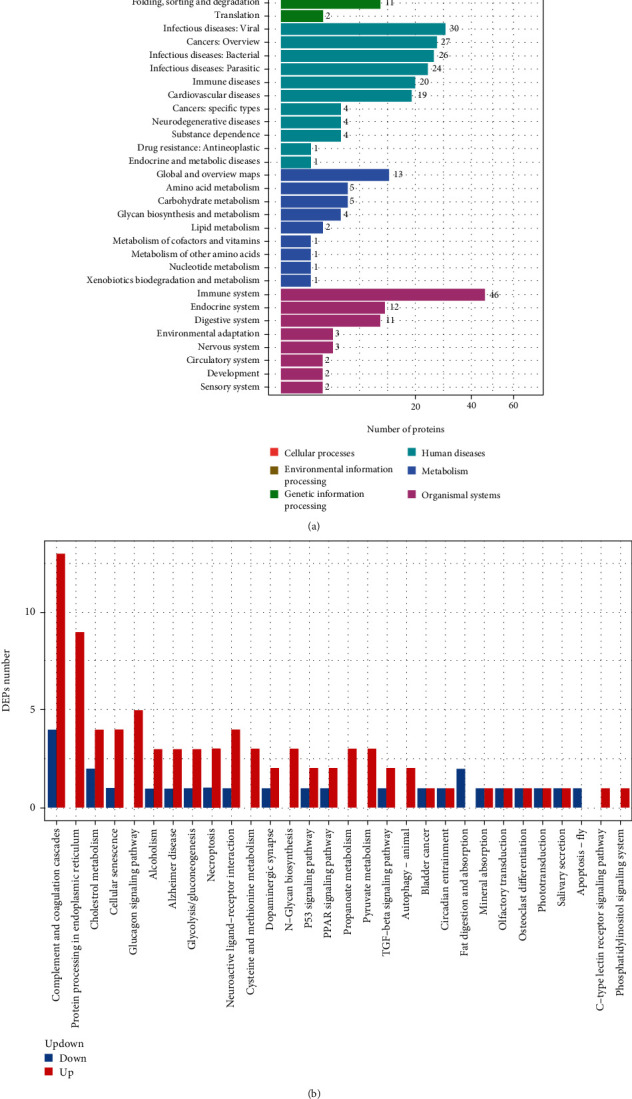
KEGG analysis of the differential expression proteins (DEPs) of severe aplastic anemia (SAA) vs. normal. (a) KEGG analysis was performed in terms of 6 catalogs including cellular processes, environmental information processing, genetic information processing, human diseases, metabolism, and organismal systems. (b) The graph showed the KEGG pathway that the DEPs enriched.

To better understand the role of the identified dysregulated proteins in SAA, we performed KEGG pathway enrichment analysis based on the identified protein markers in SAA patients and normal controls. As shown in Figure 4(a), the analysis was performed in terms of 6 catalogs including cellular processes, environmental information processing, genetic information processing, human diseases, metabolism, and organismal systems. It was obvious that the DEPs were enriched in organismal systems, particularly in the immune system. According to the classification of human diseases, DEPs could be enriched to 20 kinds of immune diseases. Above analysis results were consistent with the conclusion that SAA was an immune-mediated disease. Besides, parts of DEPs were related to infectious diseases, such as viral and bacterial diseases. In terms of cellular processes and environmental information process, the dysregulated proteins were involved in transport and catabolism and signal transduction, respectively. Specifically, Figure 4(b) showed that most DEPs were involved in complement and coagulation cascades, protein processing in endoplasmic reticulum, cholesterol metabolism, cellular senescence, and necroptosis. Notably, most of which were upregulated.

## 4. Discussion

Severe aplastic anemia is a rare disease characterized by pancytopenia and bone marrow failure; the pathogenesis of which is closely related to autoimmune T cell hyperfunction [3]. Bone marrow transplantation remains the first therapeutic choice for young patients with a matched sibling donor. Immunosuppressive therapy of ATG and CsA, with or without the thrombopoietin receptor agonist eltrombopag, are considered the standard of care in older patients and a therapeutic option for younger patients without a matched sibling donor [2, 3]. The pathogenesis of SAA is known to be closely related to abnormal cellular immunity caused by increased CD8+ T lymphocytes. Previous studies suggest that excessive secretion of Th1 type cytokines, such as interferon-*γ* (IFN-*γ*) and interleukin-2 (IL-2), leads to CD8+ cytotoxic T lymphocytes being extremely increased in patients with SAA [4]. In addition, our previous results demonstrated that both the numbers and function of myeloid dendritic cells (DCs) were significantly increased in the peripheral blood from individuals with severe aplastic anemia [5]. Moreover, it has confirmed CD34+ progenitor cells to be more apoptotic in the bone marrow of AA patients when compared with normal donors. Fas, one of the receptors in the death receptor signaling pathway, on AA progenitor cells were enhanced by IFN-*γ* and TNF-*α*, which were constitutively secreted by activated T-effector cells [6, 7]. Clinically, it is important to identify biomarkers to assess the immune status of SAA patients and to assist in diagnosis and treatment. Here, we performed a proteomic analysis to compare the plasma proteins between SAA patients and normal controls in an attempt to find suitable biomarkers to clarify the mechanism and assist the diagnosis.

Proteomics has been demonstrated as an efficient tool in clinical studies of diseases, including SAA. Liu et al. compared the proteomics of mDCs in SAA patients between controls through two-dimensional gel electrophoresis and analyzed the data using MALDI/TOF mass spectrometry; thus, they identified differentially expressed protein which might be an antigen to activate mDCs in SAA [8]. Qi et al. identified the protein of CD34+ cells from SAA patients by iTRAQ labeling combination of multidimensional liquid chromatography and tandem mass spectrometry; the results may reveal the pathogenesis of SAA, including hyperfunction of immune responses and excessive apoptosis of CD34+ cells [9]. Our present study applied DIA technology to discover dysregulated proteins in plasma of SAA patients. In recent years, DIA is an emerging quantitative proteomics approach, which combines the breadth of protein identification and reliable repeatability provided by DDA. DIA identified up to 89% of the proteins detected in a comparable DDA experiment while providing reproducible quantification of over 85% of them [10]. It reveals that this strategy is a good choice for proteomic study. DIA technology is now widely used in various fields of life science, especially in the study of tumor diseases and infectious diseases [11, 12].

Our study only included SAA patients due to immune attack mediated by T cells and excluded SAA patients with other etiologies and other autoimmune diseases. Compared to the normal control, 154 DEPs were found in the plasma of SAA patients using DIA technology. 96 proteins were upregulated, and 58 proteins were downregulated. GO analysis indicated the DEPs were mainly involved in cellular process, biological regulation, metabolic process, and immune system process, and their functions are mainly related to binding and catalytic activity, molecular function regulator, structural molecule activity, transporter activity, signal transducer activity, and transcription regulator activity. KEGG analysis showed that the DEPs were enriched particularly in the immune system; 20 proteins were involved in immune diseases. Besides, parts of DEPs were related to infectious diseases, such as viral and bacterial diseases. In terms of cellular processes and environmental information process, the dysregulated proteins were involved in transport and catabolism and signal transduction, respectively. Notably, we showed that expression of CCL18 and matrix metalloproteinase-3 (MMP3) increased in the plasma of SAA patients, while the expression of HRG and Lf was reduced.

The mature CCL18 protein consists of 69 amino acids and shares 60% sequence homology with CCL3. In vivo, the main producers of CCL18 are a broad range of monocytes/macrophages and DCs. It is interesting that CCL18 is constitutively present at high levels in human plasma and likely contributes to the physiological homing of lymphocytes and DCs and to the generation of primary immune responses [13]. More and more studies noted the critical role of chemokine in the pathogenesis of immune and inflammation diseases. DCs are key regulators in immune responses, capable of priming naive T cells. The specific expression of CCL18 preferentially attracting naive T cells may be one of the mechanisms used by DCs to interact preferentially with unprimed T cells and is likely to be an important first step in the initiation of an immune response [14]. Moreover, CCL18 production is enhanced by allergen exposure in vitro and in vivo. In turn, CCL18 inhibits the production of Th2 cytokines in CD4+ cells induced by allergen-pulsed DCs [15]. In our study, the expression of CCL18 was higher in the plasma of SAA patients. Our previous studies have demonstrated that both immature and activated myeloid DCs increased in the bone marrow of SAA patients, and the balance of DC subsets shifted from the stable form to active one, which might promote Th0 cells to polarize to Th1 cells and cause the overfunction of T lymphocytes, leading to hematopoiesis failure in SAA [16]. We hypothesized that a certain microbial infection may lead to increased levels of CCL18 in DC cells in SAA patients, attracting T cells to initiate an immune response. In addition, CCL18 inhibits Th2 cell production, which is a benefit to stimulate CTL production. Therefore, we suppose that CCL18 plays a role in the activation of T lymphocytes by DCs and participate in the immune pathogenesis of SAA.

Matrix metalloproteases (MMPs) is a group of proteases that takes part in many physiological and pathological processes of the body. Their effect involves the regulation of immune system and inflammatory cascade and breakdown of the extracellular matrix, which mainly provide ways for recruitment of immune cells and remodel the new extracellular matrix [17]. MMP3, also known as stromelysin-1, is a proteinase synthesized and secreted by synovial fibroblasts and chondrocytes in the joints; it is actively involved in joint destruction in rheumatoid arthritis patients. Therefore, it has been suggested that MMP3 was a biomarker of rheumatoid arthritis, which could reflect the severity of the disease [18]. Moreover, some research showed that MMPs may also show antiviral activity by their intracellular actions. MMP-3 exerted an antiviral effect in RAW264.7 macrophages infected with Dengue virus. Its transcription and cytoplasmic levels were upregulated in association with the elevation of antiviral cytokines. MMP-3 then translocated to the nucleus where it activated the NF-*κ*B p65 subunit; thus, this process promoted the antiviral immune response. Meanwhile, NF-*κ*B exerted a positive feedback effect as it enhanced the gene expression of MMP-3 [19, 20]. SAA is a disease in which hyperfunction T lymphocytes attack hematopoietic stem cells. The etiology of SAA is unknown, and it may be caused by a certain viral or bacterial infection. It was speculated that the elevation of MMP3 in SAA might relate to some infection. Because of continued infection, the anti-infection immunity was enhanced by the increase of MMP. Then, continuous activation of the immune system may lead to abnormal autoimmune mechanism in SAA. However, the pathogenesis of MMP in SAA should be further studied. MMP3 is expected to become a biomarker in the plasma of SAA patients.

In our cohort, circulating HRG levels were decreased in SAA patients at diagnosis. The primary structure of human HRG is predicted to be a 507 amino acid multidomain polypeptide consisting of two cystatin-like regions at the N-terminus, a histidine-rich region flanked by proline-rich regions, and a C-terminal domain, which allows the molecule to interact with many ligands, including heparin, phospholipids, plasminogen, fibrinogen, immunoglobulin G, complement complements, Fc*γ*R, and Zn^2^(+) [21]. Because of its binding profile, HRG has been suggested to play important roles in infection disease defense, the clearance of apoptotic and necrotic cells, the modulation of coagulation/fibrinolysis, and regulation of the status of immune cells [22, 23]. HRG induced shape change of human neutrophils and prolongs the survival time by binding with CLEC1A receptor on neutrophils. This combination could induce the phagocytic activity of human neutrophils against pathogen [24]. Moreover, plasma HRG was markedly decreased in septic patients, especially lower in nonsurvivors. Thus, HRG could be a biomarker for sepsis [25]. In addition, HRG significantly enhanced NK cell cytotoxicity by increasing the release of granzyme B and decreasing NK cell surface PD-1 expression [22]. The decrease of HRG in SAA may indicate the persistence of infection and would be related to poor prognosis. Infection may lead to dysfunction of neutrophils, NK cells, and other initial immune cells and then induce a series of immune system abnormalities in SAA. In addition, the decrease of HRG could lead to the insufficient clearance of apoptotic cells, which results in breaking the immune tolerance and inducing autoimmune diseases.

Human Lf is a single polypeptide chain constituted of 691 amino acids. It is an 80 kDa glycoprotein which is able to bind two ferric ions per molecule. Lf is a key element of host defenses, which is a multifunctional cationic glycoprotein secreted by exocrine glands and neutrophils. It plays important roles in the regulation of iron absorption, and the modulation of immune responses, and it has antimicrobial, antiviral, antioxidant, anticancer, and anti-inflammatory activities [26]. As shown in in vitro studies, Lf may act as a chemoattractant for immune cells, not only polymorphonuclear leukocytes but also antigen-presenting cells, such as monocytes and DCs, for which it also plays a role in activation and maturation. We speculated that the decrease of Lf level in SAA patients might be correlated with infection. The decrease of Lf may lead to the reduction of anti-infection ability and leukocyte chemotactic function, which may be involved in various microbial infections in SAA patients. Dietary Lf is able to influence systemic immune responses through the modulation of cytokine expression in the blood and lymph, including IL-4, IL-2, IL-12, and interferon-*γ*. The modulation of cytokine expression by dietary Lf could affect the Th1/Th2 balance, thus, orienting Th responses toward anti-inflammatory or proinflammatory responses [27]. In particular, in the context of aseptic inflammation, such as anemia of inflammation, preterm delivery, Alzheimer's disease, and type 2 diabetes, Lf administration reduces local and/or systemic inflammation [28]. Lf supplementation may be an option for SAA treatment, since Lf could increase the anti-infection ability of SAA patients and regulate the balance of Th1/Th2 cells and change the immune state in SAA patients.

Liquid biopsy, which detects circulating biomarkers in the blood, is noninvasive and convenient compared with traditional biopsy, and provides a new method for disease diagnosis and treatment. Along with technological advances, liquid biopsy is not only the detection of gene-based biomarkers such as cell free DNA (cfDNA) but also extended to cell-based or protein-based biomarkers, such as circulating endothelial cells (CECs), microparticles (MPs) and extracellular vesicles (EVs) [29]. Currently, liquid biopsy has been applied in the clinical management of blood diseases, especially in malignant diseases. However, there are a few relevant studies in AA. An earlier article showed that circulating membrane-derived procoagulant MPs was elevated in AA patients with a PNH clone, which could be a biomarker of thrombotic risk [30]. Albitar et al. found that the mutation profile from peripheral blood cfDNA were consistent with DNA from bone marrow cells, and peripheral blood cfDNA can serve for mutations detection [31]. By measuring circulating microRNAs in plasma, 3 plasma biomarkers were identified in aplastic anemia, in which miR-150-5p, miR-146b-5p, and miR-1 can be useful for diagnosis and monitoring [32]. Another study from the same research center confirmed that circulating exosomal microRNAs could be applied for differential diagnosis of AA and myelodysplastic syndrome [33]. Our study investigated the proteomics of plasma from AA patients using DIA technology, which also belonged to liquid biopsy in a broad sense. In the future, novel biomarkers and combinations of different types would provide a more accurate and personalized basis for disease management.

## 5. Conclusions

In conclusion, DIA is an efficient and sensitive proteomic detection method, which can be used to screen biomarkers for diagnosis and monitoring of disease. Our DIA result confirmed the immune pathogenesis of SAA. It also suggested that pathogenic infections may play an important role in the activation of the immune system and induction of immune tolerance in the early stage of SAA. CCL18, MMP3, HRG, and Lf may play an important role in the pathogenesis of SAA, which might be potential biomarkers for SAA. Exploring these new biomarkers is a benefit for the diagnosis, treatment, monitoring, and prognosis of SAA.

## Figures and Tables

**Table 1:** Characteristics of SAA patients.

Case	Age/sex	WBC (^∗^10^9^/L)	ANC (^∗^10^9^/L)	Hb (g/L)	PLT (^∗^10^9^/L)	RET# (^∗^10^9^/L)	PNH clone	Abnormal chromosome	Therapy
1	46/M	2.16	0.38	72	16	6.6	Absence	Absence	ATG+CsA
2	35/M	1.3	0.6	54	8	7.7	Absence	Absence	ATG+CsA
3	75/F	1.65	0.13	90	38	8.7	Absence	Absence	ATG+CsA
4	43/M	2.39	0.27	77	13	12.6	Absence	Absence	ATG+CsA
5	65/F	1.53	0.01	80	74	2.7	Absence	Absence	ATG+CsA
6	50/F	2.2	0.01	78	4	5.1	Absence	Absence	ATG+CsA
7	55/F	2.64	1.09	68	7	10.9	Absence	Absence	ATG+CsA
8	74/M	1.49	0.3	89	13	6.3	Absence	Absence	ATG+CsA

## Data Availability

The data used to support the findings of this study are available from the corresponding author upon request.
